# Mandibular involvement of plasmacytoma – Uncommon case report of rare entity

**DOI:** 10.1016/j.amsu.2019.07.021

**Published:** 2019-07-13

**Authors:** Vladimir Popovski, Suzana Dvojakovska, Alberto Benedetti, Goran Panchevski, Aleksandar Stamatoski, Vesna Janevska

**Affiliations:** a Clinic for Maxillofacial Surgery, Faculty of Dental Medicine, Ss Cyril and Methodius University of Skopje, Skopje, Macedonia; b Institute of Pathology, Faculty of Medicine, Ss Cyril and Methodius University of Skopje, Skopje, Macedonia

**Keywords:** Solitary plasmacytoma, Case report, Multiple myeloma, Mandible

## Abstract

Solitary plasmacytoma (SP) is an early-stage plasma cell malignancy that is in between monoclonal gammopathy of undetermined significance (MGUS) and multiple myeloma (MM) along the spectrum of plasma cell disorders. SPs can be divided into 2 groups according to location: solitary bone plasmacytoma (SBP) – these occur most commonly in the vertebrae and secondarily in long bones, and extramedullary plasmacytoma (EMP) – these encompass all nonosseus SPs. The etiology is still unknown, and they generally display a destructive course with preponderance of males. Both lesions present a risk of progression to multiple myelom. Its presence in jaws is extremely rare and when it is seen, angulus and ramus mandible are most common sites of occurrence. Histopathological examination and systemic investigation are mandatory for confirmation but support of immunohistochemistry positivity for CD138 was also done for establishing the final diagnosis.

## Highlights

• Solitary plasmacytoma of the jaw is a rare condition, especially in mandible.
• Symptoms may be underestimated because they are considered nonspecific.
• For jaw lesions it may be difficult, using routine hematoxylin and eosin stains, to determine whether the lesion is a neoplasm of plasma cells or an inflamatory lesion.
• Immunoperoxidase techniques can be helpful in making the distinction between inflammatory and neoplastic lesions.
• Plasma cell neoplasms demonstrate monoclonal light chains within the cytoplasm of plasma cells.

## 1 Introduction

The plasmacytoma is a neoplastic and monoclonal proliferation of plasma cells that usually arises within bones. Most of the lesions present centrally within a single bone, and it occurs most frequently in the spine, vertebrae, femur, and pelvis [1,2]. Infrequently, it is seen in soft tissue, in which case, the term extramedullary plasmacytoma is used. The upper respiratory tract, especially the nasal cavity, oropharynx, nasopharynx, and sinuses, is frequently involved, and it has a longer survival rate [3,4]. However, the extramedullary plasmacytoma can convert to plasmacytoma of bone and myeloma, (less than a 10% chance) both of which are associated with a poorer prognosis [5,6]. The male to female ratio of solitary plasmacytoma is approximately 2: 1, with an average age of 55 years [5]. Approximately 12%–15% of solitary plasmacytomas of the bone occur in the jaw and they are commonly involved in the posterior body of the mandible [[1], [2], [3], [4]]. On radiographic study these plasmacytoma's appear as multilocular radiolucency without any reactive bone formation [5,6]. Radiation therapy, radical extensive surgery or a combination of both is recommended as primary treatment [7].

This case has been reported in line with SCARE (Surgical case report) criteria [8].

## 2 Presentation of case

A 45-year-old patient **non smocer reported to our department**, due to a marked cyst like formation in the mandible on the left side, on a routine radiographic imaging. The patient has no subjective problems, likewise no macroscopic changes of the mandible mucosa and vestibule on the left site was note. **The lymph nodes were not palpable.**

The provisional and differential diagnosis considered then were, ameloblastoma, lymphoma, odontogenic myxoma, peripheral neuroectodermal tumor, central giant cell granuloma, Ewings sarcoma, odontogenic keratocyst, rhabdomyosarcoma and neuroblastoma. Aspiration biopsy was non-productive. Incisional biopsy was performed by senior surgeon (SD and GP), and profuse intraoral bleeding wasn't noted. Histopathology revealed atypical plasmacytoid cells with hyperchromatic nucleus.

There is no relevant psychosocial or family history. On anamnesis and physical examination, the patient reported no pain. In addition, intraoral examination revealed no extensive mass of mucosa-like color. No punctum, discharge or sinus noted.

**Fig. 1:**
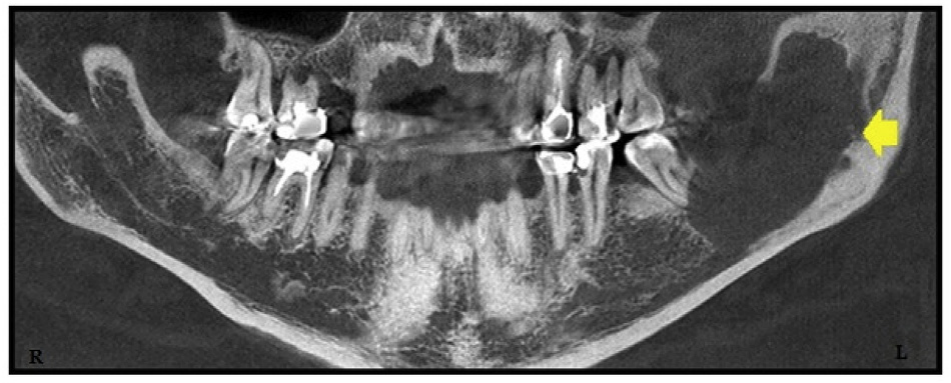
Panoramic **view showing** a large unilocular cystic lesion on the left side of mandible.

**Fig. 2:**
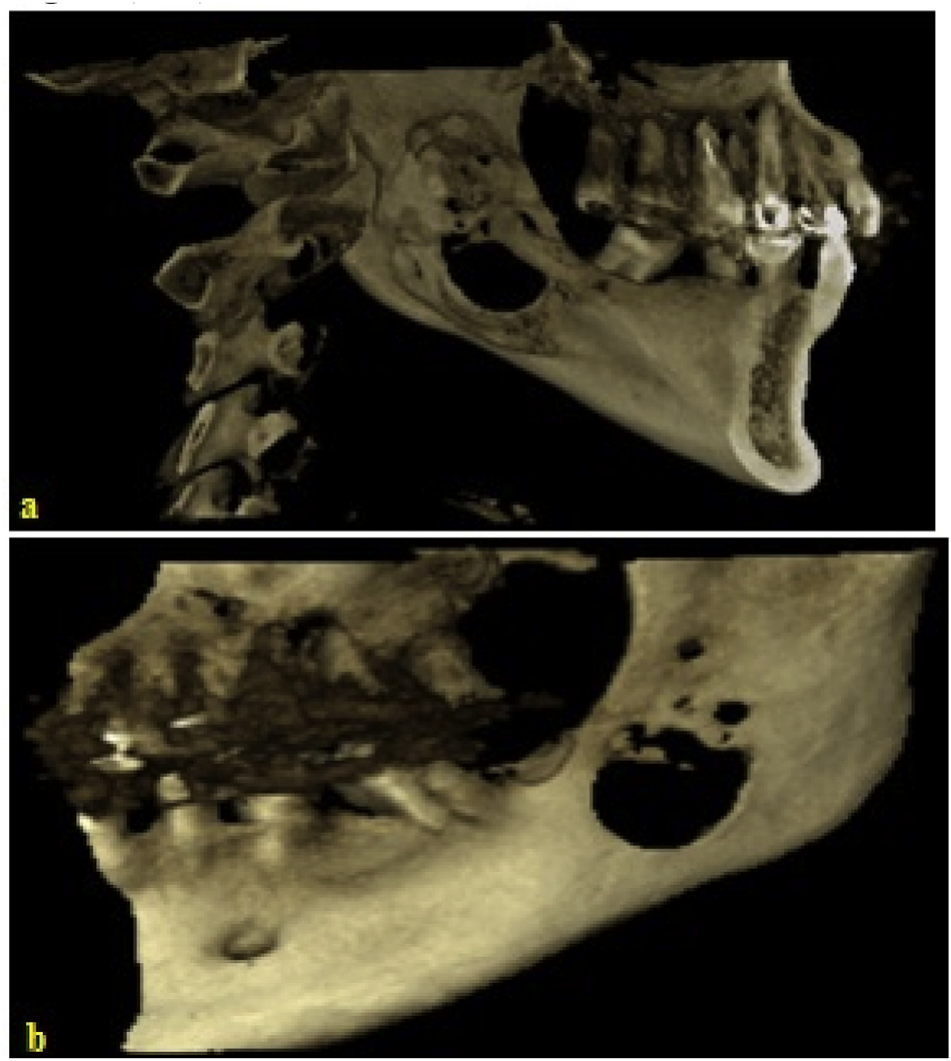
(a,b) 3D CT of the of the mandible.

On 3D radiography, a multilocular formation with sharp defined lower margin is recorded. Medial and distal boundaries were not strictly defined. Distally this formation reaches up to the middle of the mandible ramus with visible osteolysis in the middle, as well as resorption of distal root of the tooth N = 37, which was in its lumen (Fig. 1, Fig. 2).

Under general anesthesia the whole **cyst-like** formation was radically removed as well as tooth, and sent for pathohistological evaluation.

Microscopically was identified neoplastic cells proliferation constructed of cells with plasmacytic differentiation. Most of them were with abundant eosinophilic cytoplasm, eccentrically placed nucleus with granulated radial chromatin, as well as cells with depleted nuclear atypia, hyper chromic nucleus, binucleated cells and cells with vesicular nucleus with barely recognizable nucleolus. Such nodular infiltrates are separated by expressed collagen matrix, with blood vessels and older and fresh bleeding parts. Fragments of necrotized bone are also found in several focuses. Peripherally, there was population from small lymphoid cells, which have an aspect of the reactive lymphocyte component.

**Fig. 3:**
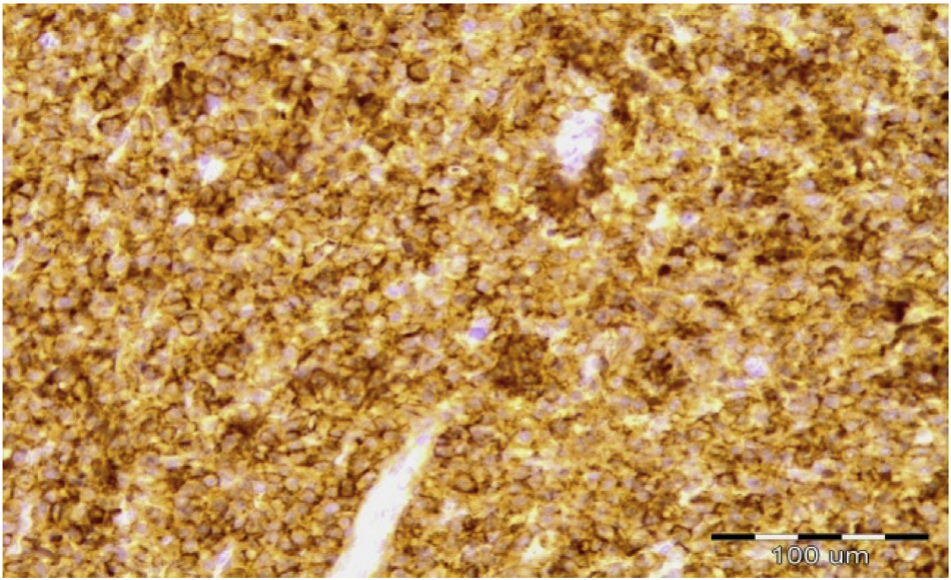
Hystopathology – plasma cells with CD 138 immunihystochemistry (×400).

With immunohistochemistry analysis neoplastic cells showed a proliferative index (Ki - 67) of 10–15%, with diffuse positivity for CD 138, MUM1 (over 70%), CD57 (less than 5%) and CD 56 (around 20–30%). The bcl1 staining showed positivity of about 70–80% of cellular populations. LCA and CD79 alpha staining showed reactive B-lymphocytic population mainly peripherally from the described plasma cells infiltrates with the presence of lymphocytes between plasma cells. Obtained result from pathological examination was plasmacytoma (Fig. 3). When the result was pathologically confirmed, the patient performed a complete radiographic evaluation and no changes were found on the skeleton that could associate with multiple myeloma.

Because the SP is radiosensitive, the patient postoperatively was exposed to radiotherapy (total dose of 34 Gy in 2 Gy fractions, 17 days). The patient's condition is regularly monitored by x-rays images. **Postoperative period was uneventful**. **Antibiotic therapy was given for 7 days.** At 3 **weeks post op period a good healing was noted.** 4 years postoperatively, there is no evidence of relapse, i.e. extramedullary plasmacytoma, or multiple myeloma.

## 3 Discussion

For a diagnosis of SP, the International Myeloma Working Group (IMWG) requires all four of the following criteria be met: biopsy-proven lesion, normal bone marrow, normal skeletal survey and absence of end-organ damage.

Оn plain radiographs, SBP can looks like some odotongenic tumors with multilocular or unilocular appearance with well-defined borders. They are commonly seen in adults in the second to fourth decade.

In our case patient he has not any symptom which would be associate on SBP. On 3D the x-ray image, there was not any sharp margins (only lower border of the formation was clearly defined) and the multilocular radiolucency was not so clear.

Primary plasmacytoma, whether osseous or nonosseous, is distinguished from multiple myeloma by the absence of hypercalcemia, renal insufficiency and anemia, normal skeletal survey, absence of bone marrow plasmacytosis, and serum or urinary paraprotein level of less than 2 g/dL.

In multiple myeloma neoplastic proliferation of highly specialized B-lymphocytes produces a single immunoglobulin. So, it's characterized by the disseminated monoclonal overproduction of immunoglobulin's in blood and/or by detecting Bence Jones proteins in urine. In our case, in Complete Blood Count test (CBC and Diff test), white blood cell (WBC: 5,3 × 10∧9/L), red blood cell (RBC: 5,4 × 10∧12/L), hemoglobin (Hb: 15.3 g/dL), hematocrit (HCT: 46.2%) and platelet (236000/mcL) were in the normal range. In biochemistry test, urea (4,95 mmol/L) and creatinine (76 μmol/L) were normal and hypercalcaemia was not found so renal insufficiency was ruled out. Serum immunoelectrophoresis was not showed an increase in M-protein [immunoglobulin (IgG) type]. Clonal plasma cells involved in plasmacytoma frequently produce a monoclonal immunoglobulin as well as κ or λ light chains. Here, in our analysis, kappa and lambda light chains staining showed emphasized “edge “artifact, however, the prevalence of lambda positivity in the neoplastic cells is evaluated, while the kappa staining was part of the reactive lymphocytes.

Bone marrow biopsies are performed to ensure the disease is localized. In solitary and extramedullary plasmacytoma, there will not be an increase of monoclonal plasma cells in bone marrow [ [9,10]]. In our case bone marrow biopsies were negative.

All patients with plasmacytomas require follow-up for at least the first five years after treatment has been completed.

## 4 Conclusion

Solitary bone plasmacytomas rarely occur in maxillofacial areas affecting mandible. In this case the patient was referred for complete treatment which includes radiation therapy and radical extensive surgery. Continuous follow-up of patient is imperative. Chemotherapy should be reserved for those cases progressing to multiple myeloma.

## Consent

Written informed consent was obtained from the patient for publication of this case report and accompanying images. A copy of the written consent is available for review by the Editor-in-Chief of this journal as required.

## Conflicts of interest

All authors declare that they have no competing interests.

## Provenance and peer review

Not commissioned, externally peer reviewed.

## Ethical approval

There is no ethical approval because it is not research study. We have the written consent of patient to published this case report. All applicable international, national, and/or institutional quidelines for the care and use of animals or human participans were in accordance with the ethical standards of institution or practice at which the studies were conducted.

## Sources of funding for your research

None of the author.

## Author contribution

1. Dr.Vladimir Popovski– - Study concept or design, data collection, literature search, writing paper, final decision to publish.
2. Dr.Vladimir Popovski, Dr Alberto Benedetti, Dr Aleksandar Stamatoski-literature search, final decision to publish.
3. Dr. Suzana Dvojakovska- Supervised the writing of the manuscript, final decision to publish.
4. Dr. Goran Panchevski: Supervised the writing of the manuscript, final decision to publish.
5. Dr.Vesna Janevska: Supervised the writing of the manuscript, final decision to publish.

## Conflicts of interest

None of the author.

## Registration of research studies

This paper is only medical case report.

## Guarantor

Vladimir Popovski.
